# ‘If I had not taken it [HIVST kit] home, my husband would not have come to the facility to test for HIV’: HIV self-testing perceptions, delivery strategies, and post-test experiences among pregnant women and their male partners in Central Uganda

**DOI:** 10.1080/16549716.2018.1503784

**Published:** 2018-08-09

**Authors:** Joseph K. B. Matovu, Rose Kisa, Esther Buregyeya, Harriet Chemusto, Shaban Mugerwa, William Musoke, Caroline J. Vrana, Angela M. Malek, Jeffrey E. Korte, Rhoda K. Wanyenze

**Affiliations:** a Department of Disease Control and Environmental Health, Makerere University School of Public Health, Kampala, Uganda; b Directorate of Research and Strategic Information, Mildmay Uganda, Kampala, Uganda; c National Disease Control, Ministry of Health, Kampala, Uganda; d Department of Public Health Sciences, Medical University of South Carolina, Charleston, SC, USA

**Keywords:** Pregnant women, HIV self-testing, male, sexual partners, Uganda

## Abstract

**Background**: HIV self-testing (HIVST) can improve HIV-testing rates in ‘hard-to-reach’ populations, including men. We explored HIVST perceptions, delivery strategies, and post-test experiences among pregnant women and their male partners in Central Uganda.

**Methods**: This was a qualitative study implemented as part of a pilot, cluster-randomized oral HIVST intervention trial among 1,514 pregnant women attending antenatal care services at three health facilities in Central Uganda. The qualitative component of the study was conducted between February and March 2017. We conducted 32 in-depth interviews to document women and men’s perceptions about HIVST, strategies used by women in delivering the kits to their male partners, male partners’ reactions to receiving kits from their female partners, and positive and negative social outcomes post-test. All interviews were audio-recorded, transcribed verbatim, and analyzed manually following a thematic framework approach.

**Results**: Women were initially anxious about their male partners’ reaction if they brought HIVST kits home, but the majority eventually managed to deliver the kits to them successfully. Women who had some level of apprehension used a variety of strategies to deliver the kits including placing the kits in locations that would arouse male partners’ inquisitiveness or waited for ‘opportune’ moments when their husbands were likely to be more receptive. A few (three) women lied about the purpose of the test kit (testing for syphilis and other illnesses) while one woman stealthily took a mucosal swab from the husband. Most men initially doubted the ability of oral HIVST kits to test for HIV, but this did not stop them from using them. Both men and women perceived HIVST as an opportunity to learn about each other’s HIV status. No serious adverse events were reported post-test.

**Conclusion**: Our findings lend further credence to previous findings regarding the feasibility of female-delivered HIVST to improve male partner HIV testing in sub-Saharan Africa. However, support for women in challenging relationships is required to minimize potential for deception and coercion.

## Background

A growing body of literature on oral HIV self-testing (HIVST) suggests that it can help to reach people that were not previously targeted by conventional HIV-testing approaches, including male partners and couples [1–3]. A recent systematic review and meta-analysis that examined the effects of HIVST compared with standard HIV-testing services found that HIVST not only doubled uptake of testing among men but also improved HIV-testing frequency particularly among men who have sex with men [4]. Studies conducted in Kenya [2,3,5] and Malawi [1,6] suggest that distribution of HIVST kits to male partners through female partners is an acceptable approach that provides an opportunity for men to test for HIV without necessarily traveling to a health facility. This may overcome the barriers related to lack of time or traveling to a health facility (which men presume to be a women’s preserve) to test for HIV [7,8]. Thus, the delivery of HIVST kits to the male partners at home can help to improve HIV-testing rates among men.

Previous efforts to improve male partner HIV testing through female-delivered invitations for men to test for HIV at antenatal clinics (ANC) have yielded conflicting results [9,10]. In most studies, less than 20% of men invited to ANC honored such invitations, with 20–40% of those honoring the invitations testing for HIV [10,11]. These findings suggest a need for alternative HIV-testing approaches that can help to improve HIV-testing rates among men, including female-delivered HIVST. However, while previous studies have reported impressive HIV-testing uptake rates among male partners and couples following female-delivered HIVST [2,3], only one study has explored the strategies that women used to deliver HIVST kits to their male partners [5]. Even then, this study did not document men’s reactions to receiving kits from their female partners. Given the gender imbalance between men and women particularly in sub-Saharan African countries – where men take the upper hand and control decision-making processes in the home [12] – the idea of using women to introduce HIVST kits to male partners can have adverse consequences for the women. This is particularly the case when such women deliver the kits without seeking the male partner’s consent first. Understanding how the women have been able to successfully deliver the kits to their partners and convince them to test could further enhance the support for other women if this approach is adopted as a model for partner notification and testing.

In this paper, we describe women and men’s perceptions of HIVST as an HIV-testing strategy; strategies used by women in delivering the kits to their male partners; how women managed to convince their male partners to use the kits; and women and men’s post-test experiences, including any positive and negative social outcomes post-test.

## Methods

### Study site

This qualitative study was conducted as part of a pilot, cluster-randomized HIVST intervention trial whose primary objective was to improve HIV-testing rates among male partners of women attending ANC services at three health facilities in Central Uganda (Nakaseke Hospital, Mpigi Health Center IV, and Entebbe Hospital). The three health facilities are all public health facilities that were supported by Mildmay Uganda (MUg), one of the largest HIV service agencies in Uganda. In the year preceding the intervention, Entebbe Hospital registered 7,034 pregnant women attending their first ANC visit; of these, only 6% (466) had their male partners tested for HIV. Mpigi HCIV served 3,245 pregnant women at first ANC, and 3.1% (102) had their male partners tested for HIV. On the other hand, Nakaseke Hospital provided care to 1,523 pregnant women at first ANC, and 31.5% (480) had their male partners tested for HIV.

### Overview of the HIVST intervention

The intervention trial aimed to improve male partner HIV testing through female-delivered oral HIVST (OraQuick^©^ HIV self-test, OraSure Technologies, Inc., Bethlehem, PA). A total of 1,514 pregnant women were enrolled into the study; 777 in the intervention arm and 737 in the control arm. Women in the control arm received general health information about the importance of male partner HIV testing and were asked to encourage their male partners to test for HIV at the participating health facilities. Women in the intervention arm received two to four oral HIVST kits; one for themselves, one for their male partners, and two for any other adults in the household. Intervention women received instruction on how to deliver the kits to their male partners, how to perform the HIV self-test (using written, pictorial materials and a demonstration video), and how to read and interpret HIV test results. Women in the intervention arm also discussed and role-played the different ways in which they could deliver the kits to their male partners and interest them to use the kits. As part of the intervention, women and their male partners (in either arm) were interviewed at baseline and followed up at month 1 and month 3 post-baseline. At the end of the intervention, purposely selected women and men who received and used the oral HIVST kits during the intervention period (see ‘Participant selection’ below) were invited to participate in this qualitative study.

### Study design and population

This qualitative study was conducted among pregnant who were given HIV self-test kits to deliver to their male partners and male partners who received and used them to test for HIV. The study was conducted between February and March 2018. Pregnant women who failed to deliver the kits to their male partners and male partners who refused to use them were excluded from this study since they did not have any user-experiences to share.

### Participant selection

At the end of the intervention, study interviewers were asked to identify, at each site, five to six women who had successfully delivered the HIVST kits to their male partners; and five to six men who had used the HIVST kits delivered to them by their female partners. These individuals did not necessarily have to be members of the same couple. Interviewers were instructed to include women and men in concordant HIV-positive, concordant HIV-negative, and HIV-discordant relationships in order to document the HIVST experiences of people in different HIV-status relationships. All the selected participants were asked to come to the study site for interviews, and their travel costs were reimbursed. Individuals who failed to come to the health facility as scheduled were traced and interviewed in the community using the locator information that they provided during the intervention period.

### Data-collection procedures and methods

At the end of the intervention period, in-depth interviews were conducted with pregnant women to explore the anticipated fears/barriers that they had in mind when they were asked to deliver HIVST kits to their male partners; the strategies that they *actually* used to deliver the kits to their male partners; how they managed to convince their male partners to use the kits to test for HIV; and what happened after HIVST, including any negative or positive social outcomes post-test. Similar issues were explored among male partners of pregnant women (e.g. men’s initial thoughts about HIVST) to triangulate the data obtained from female partners but also to capture their perceptions and experiences regarding HIVST. We also asked men whether or not they were comfortable receiving HIV self-test kits from their female partners. All data collected were recorded on digital audio-recorders with permission from the participants. Interviews lasted about 1–2 h.

### Data analysis

Data analysis was conducted deductively following a thematic framework approach [13]. The thematic framework approach is used for identifying, analyzing, organizing, describing, and reporting patterns (themes) found within a qualitative dataset [14]. It follows a series of eight inter-related stages, namely: (1) preparing the data for analysis by transcribing the interview; (2) becoming familiar with the interview by reviewing the data transcripts or re-listening to all or parts of the digital-recording; (3) reducing the data into themes through a process of data coding – or applying paraphrases or labels (‘codes’) that describe what the data say; (4) searching, reviewing, defining, and collating all the potentially relevant coded data extracts into categories or themes; (5) developing a working analytical framework – researchers compare the labels that they have applied to the data and agree on a set of codes to apply to all subsequent transcripts; (6) applying the analytical framework by indexing subsequent transcripts using the existing codes; (7) charting data into the framework matrix; and (8) interpreting the data [13,14].

Prior to data analysis, all digitally recorded interviews were transcribed verbatim and translated from Luganda, the language of the interview, to English. Initially, JKBM and RK manually read through 10 transcripts to identify any key issues based on the primary objective of the study; namely, to document perceptions, HIVST delivery strategies, and post-test experiences of women and men with regard to HIVST. The initial 10 transcripts were purposely selected to ensure site representation – with three transcripts selected from Nakaseke Hospital and Mpigi Health Center IV and four transcripts selected from Entebbe Hospital. The process of selecting the initial transcripts also took into consideration participants’ HIV status and the ease with which pregnant women delivered the kits to their male partners. Transcripts for male partners were purposely matched with those of their female partners to identify any emerging patters in the experiences shared, e.g. how long it took a woman to deliver the kit to her male partner (as reported by herself) and what the male partner reported with regard to how soon they received the kit from their female partners.

We read through the initial transcripts with the primary objective of the study in mind, and all emerging issues were coded as either belonging to ‘perceptions’, ‘strategies’, or ‘post-test experiences’ through a process of constant-comparison and consensus-building. Each issue was assigned a code; and using these codes, we reviewed the remaining 22 transcripts to identify perceptions, strategies, or post-test experiences pertaining to HIVST, while noting down any new issues along the way. At the end of the review, different emerging issues (subthemes) were categorically grouped under different a priori themes, depending on the extent to which they related with each theme. There were four a priori themes, namely: (1) perceptions about HIVST as an HIV-testing strategy; (2) strategies used by women to deliver the kits to their male partners; (3) strategies used by women to encourage their male partners to use the kits for HIV testing; and (4) post-test experiences following HIVST. Study findings were reported following the consolidated criteria for reporting qualitative research (COREQ) [15].

## Results

### Characteristics of the participants

Thirty two (32) in-depth interviews (17 women and 15 men) were conducted. Table 1 shows the characteristics of the participants by study site, sex, age-group, level of education, religious affiliation, and self-reported HIV status. Of those interviewed, 10 participants were from the Entebbe site; 11 were from the Nakaseke site; while the remaining 11 participants were from the Mpigi site. Of the 32 participants, five (15.6%) were in concordant HIV-positive relationships, nine (28.1%) were in HIV-discordant relationships, and 18 (56.3%) were in concordant HIV-negative relationships. Nearly half (46.9%) of the participants were aged 25–34 years, 34.4% were aged 35 years or older, and 18.7% were aged between 18 and 24 years. Slightly more than half of the participants (53.1%) had primary education, 34.4% had secondary education, and 12.5% (only men) had post-secondary education (e.g. a university degree).

**Table 1.** Characteristics of the study participants.

Characteristic	Male (*n*)	Female (*n*)	Total (*N*, %)
**Study site**			
Entebbe Hospital	6	4	10 (31.2)
Nakaseke Hospital	4	7	11 (34.4)
Mpigi Health Center IV	5	6	11 (34.4)
**Age group**			
18–24	2	4	6 (18.7)
25–34	6	9	15 (46.9)
35+	7	4	11 (34.4)
**Highest level of education attained**			
Primary	7	10	17 (53.1)
Secondary	4	7	11 (34.4)
Postsecondary^a^	4	–	4 (12.5)
**Religious affiliation**			
Catholic	7	9	16 (50.0)
Protestant	6	5	11 (34.4)
Muslim	2	3	5 (15.6)
**Individual HIV status^b^**			
HIV-negative	11	13	24 (75.0)
HIV-positive	4	4	8 (25.0)
**Couple HIV status^c^**			
Concordant HIV-negative	8	10	18 (56.3)
Concordant HIV-positive	1	4	5 (15.6)
HIV-discordant	6	3	9 (28.1)
**When did you give the kit to your male partner? (Women only, *N* = 17)**			
Same day/next day	NA	8	8 (47.1)
Within 1 week	NA	5	5 (29.4)
> 1 week	NA	4	4 (23.5)

^a^Post-secondary education’ is used to refer to those with a diploma or university degree certificate.

^b^Self-reported HIV-status based on HIV self-testing.

^c^With the exception of two couples where both partners were interviewed, only one member of each couple was interviewed.

### Perceptions about HIVST, delivery strategies, and post-test experiences

Women and men’s perceptions about HIVST, delivery strategies, and post-test experiences have been presented separately in each of the subsections below.

#### Perceptions about HIVST as an HIV-testing strategy

Pregnant women and male partners had different perceptions about HIVST kits delivered in the home. While women were more concerned about their male partners’ reactions when they delivered the kits to them, male partners were more skeptical about whether or not the kits could *really* test for HIV. These perceptions are illustrated in the following subsections.

##### Fear about male partners’ reactions

When asked how they initially reacted to the need to take the kits to their male partners, women cited fear of partners’ reactions as the most common perception that came into their mind. Most of the women thought that their male partners would ‘ask me what I had brought home’, while others thought they would most likely refuse to use the kits. Women noted that they had previously found it hard to convince their male partners to test for HIV, and many had ever tried to encourage them to test without success. Thus, many of the women were not sure how their male partners would react to receiving the kits from them and whether or not this (taking kits home) would not be counterproductive. However, with adequate preparation, women navigated these fears and successfully delivered the kits to their male partners.

##### Skepticism about HIVST kits

In response to the question on how they felt when they were informed by their female partners about the HIV self-test kits, a majority of the male partners reported that they did not think that a kit that uses ‘saliva’ (oral mucosal transudate) would be able to test for HIV. This was largely because they could not comprehend how HIV testing could be done without using a blood sample.

… I asked myself ‘is this really true’? Can really a person just get that ‘spoon’ [the kit] and pass it on the gum and then … [he spreads his hands] and test for HIV? Still that was running in my mind – wondering … and up to now; I am still not convinced because she told me that you know the ‘saliva’ settles there and the person who is positive, there is this and that – she is not a nurse, she is … [laughs] so you have to know I am still asking myself really. (Male partner in a concordant HIV-negative relationship, 38 years, Entebbe)

These sentiments were expressed by other men who also thought that HIV testing can best be done while using blood rather than oral mucosal transudate. Thus, while these doubts did not stop men in our study from using the kits, it is likely that other men may find it difficult to believe results from HIV self-tests, calling for a need to intensify health education about oral HIVST, as this approach is rolled out in the future.

#### HIVST a key strategy to reach men

Besides the fears and concerns around HIVST, both men and women perceived HIVST as a strategy that would address men’s apparent lack of time to go to the health facilities to test for HIV. This is because the kits can be used to test for HIV in a private setting, including in people’s homes. Women particularly viewed HIVST as a strategy that would not only ‘make it easy’ for men to test for HIV but also offer them with the opportunity to know their male partners’ HIV status.

… for us pregnant women, our men find it difficult to go to a health facility because they say they are busy, they don’t get time to go to a health facility to have blood taken so that we can be tested as a couple but when I take the self-test kit at home and I explain it to him, it makes it easy for him to use it. (HIV-negative woman who tested with her HIV-negative male partner, age 25, Mpigi)

When asked whether it was of concern for them to receive the kits from their female partners, a majority of men reported that they did not mind who brought the kits home as long as there was harmony in the relationship. Indeed, with the exception of a few men who reported that such kits should rather be brought into the home by male partners, most men did not have any reservations about receiving the kits from their female partners. The men who preferred to have the kits delivered by male partners reasoned that it would give them the opportunity to test for HIV (and establish their HIV status) before they could deliver the kits home, something that would not be possible if the kits were delivered by the women.

#### Strategies used by women to deliver HIVST kits to their male partners

As shown in Table 1, eight of the 17 women (47%) interviewed reported that they delivered the kits to their male partners either on the same or the next day, five women (29.4%) delivered them within one week, and four women (23.5%) delivered them after one week.

**Figure 1.**
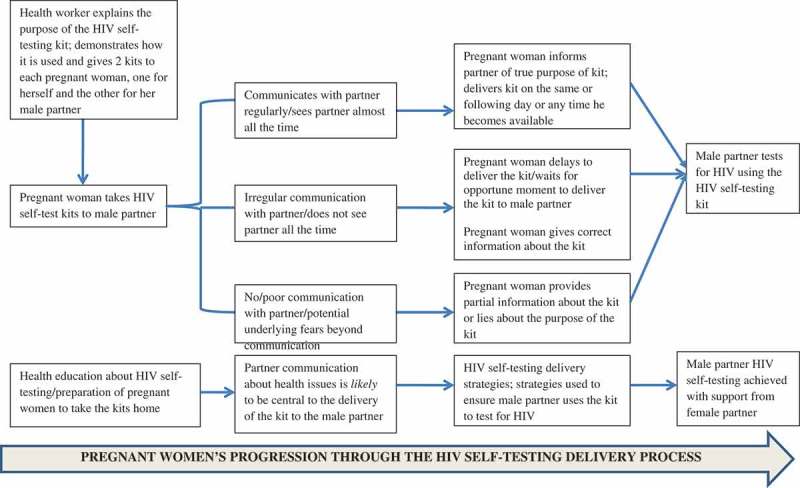
Pregnant women’s progression through the HIV self-testing delivery process.

Figure 1 presents a schematic illustration of the process that women followed in delivering the kits to their male partners and what they did to ensure that their male partners used them to test for HIV. As shown, the HIVST delivery process emerged as a function of partner communication about health issues on the one hand and presence of the male partner in the home at the time of delivering the kit on the other. For instance, women who communicated with their male partners about health issues more regularly and who were in regular contact with their male partners were more likely to deliver the kits to them on the same or the following day after obtaining them from the health facility. If their male partners were away on a trip, for instance, these women still delivered the kits to them the moment that they came back. Conversely, women who had irregular communication with their male partners delayed to deliver the kits to their male partners even if such partners were physically around and not away on any trip. The level of partner communication also seemed to influence how much information the female partners gave to their male partners regarding the kits. Women who were skeptical that their male partners would not use the kits if they told them that they (the kits) were meant to test for HIV provided partial information about the kit or lied about their purpose altogether. The strategies that women used to deliver the kits to their male partners are summarized in each of the subsections below.

##### Placing kits in visible locations

All the eight women who delivered the kits on the same or next day reported that they placed HIVST kits in visible locations to arouse inquisitiveness from their male partners. For instance, some women said that they left the kits at the bedside because they thought this would prompt the male partners to ask about ‘these things’. As expected, men inquired about the ‘things’ that were placed in visible locations, and women used this opportunity to initiate discussions around HIV testing and to explain the intended purpose of the kits.

I told him it [the kit] tests for HIV, I didn’t hide it from him, so he asked me ‘what if it turns out that I am HIV-positive, won’t I get so scared?’ So, I told him not to get scared because if it turns out that he was HIV-positive, we shall go to the hospital and be counseled. So he asked me, ‘what if I am found HIV-positive and you don’t have it or you have it [HIV] and I don’t, won’t you leave me and go?’ I told him I won’t leave you because we will not have been the first to get it [HIV]… (HIV-negative female partner in an HIV-discordant relationship, age 34, Nakaseke)

##### Waiting for ‘opportune moments’

Nine women indicated that they waited for some days (one woman said she waited for 2 weeks) to introduce the kit to their male partners and used ‘opportune moments’ to introduce HIVST kits to their partners. These women gauged their male partners’ ‘mood’ coupled with the quality of their relationships to decide when it was appropriate to deliver the test kits, e.g. in the morning or when their male partners were in ‘good moods’.

Okay, I approached him when he was in his good moods because there are times when you can approach him amidst stress and he barks at you. For this one, I cracked some jokes, and I was able to gain the courage to test him. I realized that if I was to be scared I would then have made mistakes by not doing the test as we were instructed at the health facility. (HIV-positive female partner in an HIV-discordant relationship, age 20 years, Entebbe)

#### Strategies used by women to encourage their male partners to use the kits

Upon introducing the kits to their male partners, women needed to convince them to use the kits for HIV testing. Women in good-quality relationships, characterized by frequent communication between partners about health issues, did not find it difficult to convince their male partners to test for HIV. Most of these women said their male partners accepted using the kits without a moment’s hesitation. However, women who were skeptical about their male partners’ readiness to use the kits opted to use a variety of approaches, some of which can be considered to be less honest, in order to convince their male partners to use the kits for HIV testing. These approaches are summarized in the subsequent sections below.

##### Seeking the support of health workers

Four women opted to engage the support of health workers at the facility where they received the HIVST kits in order to convince their male partners to use the kits. This was particularly the case in situations where men were initially hesitant to use the kits. In one case, a female partner used a combination of verbal threats, a phone call to the health worker to talk to the male partner, and a three-day deadline within which the male partner had to make a decision, as illustrated in the quotation below:

Mine had refused [to use the kit] and I told him that I am going to elope from the marriage ‘because you have been away for a long trip [yet] I don’t know where you have been’ and he said, ‘I cannot use those things’. I gave him about three days, we were seated there (she points to the house verandah) and I told him that I want to go back to the hospital for ANC but you are making it hard for me. He asked me how [he was making it difficult for me to go back for ANC] and I told him that ‘it’s because you refused to test [for HIV]’ … then I called the health worker on phone and I [passed on the phone] to him and he [the health worker] told him that ‘we taught your wife so she should direct you on how to use those kits’. He listened and then we entered the house and I showed him on how to use the kits and he did not find any problem with the kit. (Female partner in a concordant HIV-negative relationship, age 42, Nakaseke)

Besides engaging health workers to convince their partners to use the kits, women positioned the kit as an HIV-testing tool that was sent to the male partners by the health workers. During interviews with male partners, some men informed us that their female partners told them that the HIVST kits were sent to them by the health workers who insisted that used kits should be returned to the health facility. One HIV-positive, 40-year-old male participant in an HIV-discordant relationship from Entebbe said his female partner ‘just told me that the health providers have requested her to give it to me to use it … So, I went ahead to do what she wanted me to do and then I gave her back her stuff and she returned them back to the health facility’.

##### Lying about the purpose of the kit

Three women opted to lie about the purpose of the kit. In one case, an HIV-positive woman from Mpigi told her partner that the kit tests for ‘syphilis’ and that after performing the test, she would have to return the kits to the health workers to ‘tell me what your results are’. She reported that the man was able to use the kit as instructed and that he did not suspect that he was testing for HIV. The male partner tested HIV-positive, but she did not tell him the results immediately. Instead, she encouraged him to go for confirmatory testing which he refused to do. We learned from this woman that her partner had always said that he was HIV-negative and constantly made reference to the fact that he would dissolve their marriage if he found that his wife was HIV-positive. This woman lied to the partner about the purpose of the kit for fear that if the man tested HIV-negative (she was already HIV-positive herself), he would ask for her results, which would cause her marriage to break. So, she lied about the purpose of the kit as a way of coping with her own status coupled with her fears of the partner’s reaction if he knew of her HIV-positive status. This woman later checked her husband’s wallet and found out that he was already HIV-positive and receiving HIV treatment from another facility. This could probably explain why she did not correct the lies that she had made to her husband that the HIVST kit was meant to test for syphilis.

In another case, an HIV-positive male participant from Entebbe reported that he was told that the HIVST kit was meant to ‘check and detect any disease in my body’. This man did not get to learn about his HIV sero-positivity until after one month when he took a confirmatory HIV test at another health facility (not at the study site). Incidentally, his HIV-negative wife, who learned of her husband’s HIV-positive status through HIVST (since she knew how to interpret the HIV results), opted to keep quiet about the results while insisting that it would be the health workers to tell him what ‘kind of diseases’ the kit had detected in his body.

##### Concealing some information about the purpose of the kit

Two women opted to provide partial information about the HIVST process from their male partners. The difference between this group and the group that opted to lie about the purpose of the kit was that while these women did not lie, they just did not provide adequate information to their male partners that would have helped them to make an informed decision. One 25-year-old male participant from Entebbe said that his female partner ‘just opened the kit and gave it to me’ while emphasizing that she would take the kit back to the health workers after he had tested:

Participant: ‘Hmmm, no she did not explain to me anything but just told me that the health worker told me that you get this thing do it like this and this then I take it back [to them]’.

Moderator: ‘Ok; didn’t she tell you what it is, what it does?’

Participant: ‘No, she didn’t explain to me but depending on what I had read on the paper she had brought, I knew that it was meant for HIV testing’

In another case, a male respondent told us that his partner just told him that ‘you are going to use it [HIVST kit] the way they [health workers] have instructed me to tell you and I will do the same’; insisting that he was not told what the kit was testing for or what the results were. A few days after HIVST, his wife informed him that the health workers had invited him to go along with her to the health facility to receive the results of the test, and when he did, he was told that he was HIV-positive and initiated on antiretroviral treatment.

##### Controlling the HIVST process

Two women opted to conduct the ‘self-testing’ exercise themselves rather than leave their male partners to do so. One woman convinced her male partner that if she allowed him to do the test himself, ‘he would do it wrongly’; so, she went ahead and swabbed him and conducted the test herself. In one extreme case, a female participant swabbed her husband while he was asleep (during the day) ostensibly because she ‘did not trust him’. This participant informed us that the male partner was too tired to feel anything at first until when she begun swabbing the ‘upper gum’.

So, I did not trust him and the way I tested him, if you told it to anyone, they would not believe you. I timed when he was asleep and I swabbed with the kit down and when I was swabbing the upper gum, he woke up, and after waking up, after a little while I showed him the results. I didn’t trust him at first because these men are hard to trust …’ (HIV-negative female partner in a concordant HIV-negative relationship, age 23 years, Nakaseke)

#### Post-test experiences after HIVST

##### No serious adverse events reported

Participants were asked if they experienced any positive (e.g. increased support from the partner; pregnant women being escorted to ANC by their male partners) or negative (marital dissolution, or physical harm or threats) social consequences after performing HIVST. A few women, especially those who initially lied about the purpose of the kit, indicated that their male partners were angry (after they found out that they were testing for HIV) that their female partners had taken them through the HIV-testing process unaware. However, these largely verbal confrontations did not result in physical fights or any other form of harm. On the whole, there were no serious adverse consequences that resulted from using the kits. Instead, women reported gaining positive experiences from HIVST, including the opportunity to learn about their partners’ HIV results; improving the quality of their relationship (e.g. through improved communication) and HIV-status disclosure. Most of the women indicated that they were happy that they took the kits home because if they did not do so, there was no way they would have known their male partners’ HIV status.

… if I had not taken it [HIVST kit] home, my husband would not have come to the facility to be tested for HIV but I took it and told him that once it shows two lines, it means you have HIV and if it shows one line it means you don’t have HIV and when we came here … they drew blood from us and tested us. He was HIV-positive (which he didn’t know) and on that very day he started on medication. And if I had not taken that kit, he didn’t have the idea in his mind to come to hospital to test for HIV but the oral kit influenced him to come to the hospital so that he knows the truth about his status and after that day he started getting treatment’ (HIV-negative woman in an HIV-discordant relationship, age 34, Nakaseke)

##### HIVST facilitated HIV-status disclosure and/or support from partner

In an interesting scenario, an HIV-positive female respondent from Mpigi narrated how the presence of the HIVST kit in the home prompted her male partner to disclose his previously undisclosed HIV sero-positive status to her, despite having known his HIV sero-positive status for the past three years and was already enrolled into HIV care. This male partner refused to use the kit when it was delivered to him by his female partner. It was due to his female partner’s consistent reminders about HIV testing that he opened up; and informed her that he was already HIV-positive. Thus, the presence of the kit facilitated HIV-status disclosure in a situation where the male partner (who already knew his HIV-positive status) had opted to keep quiet about his status. In another in-depth interview with an HIV-positive female partner in an HIV-discordant relationship from Mpigi, she said that although she was scared of what her husband might do after learning that she was HIV-positive, he actually ‘did not show any anger’ and ‘since that time, he asks when am going for treatment and he gives me transport’. This female participant indicated that instead of the relationship developing cracks, her husband started to support her the moment he learned of her HIV-positive status. He supports her with money for transport to the clinic, and also reminds her of when to take her drugs to improve adherence.

## Discussion

Our study of the perceptions, HIVST delivery strategies and post-test experiences of women and men who used HIV self-test kits in Uganda shows three interesting findings: (1) there was initial fear among women of how they would introduce the kits to their male partners, and, among men; concerns about the kits yielding inaccurate HIV test results since they don’t use blood for HIV testing; (2) women used a diversity of strategies to introduce HIV self-test kits to their male partners and to encourage them to use the kits; and (3) there were no major adverse events following HIVST. Despite the initial apprehension about how to deliver the kits (among women) and the initial concerns about the ability of the test kits to yield accurate results (among men), the majority of the participants had favorable perceptions toward HIVST and shared more positive post-test experiences after performing HIVST. We also found that male partners readily accepted using the kits delivered to them by their female partners, although a few men would have preferred to test alone first (before receiving the kits from their female partners) or to deliver the kits to their female partners themselves. Either way, our findings suggest that HIVST is a feasible HIV-testing strategy that can increase male partner HIV testing in Uganda.

### Fear of partners’ reactions a key hindrance to HIVST

Our findings of the initial fears from women regarding introducing the kits to their male partners are consistent with earlier reports of fears of the consequences of introducing HIV self-test kits in the home. In a qualitative study by Matovu et al. [16], both men and women were concerned that introducing the kits in the home would result in dire consequences for the couple, particularly to the HIV-positive partner in the relationship. These consequences included fears of marital disruption and suicidal ideation [17] due to the absence of pre- or post-test counseling. Similar fears have been documented in other studies [18,19] especially with regard to fears of what could happen in the home in the event that one of the partners is HIV-positive, in the absence of post-test counseling. To deal with these fears, we provided adequate preparation to improve the women’s technical competencies and negotiation efficacy to improve their ability to deliver the kits as well as encourage their partners to test alone or together with them. As a result, women were able to overcome the initial fears and successfully delivered the kits to their male partners with no serious adverse events reported. Indeed, a review of the evidence of harm from HIV self-tests found that although the potential for harm is discussed in the literature on self-tests, there is very little evidence that such harm occurs [20]. Collectively, our findings and findings from previous studies suggest that women can safely deliver HIV self-test kits to their male partners, and this, in itself, can help to improve HIV-testing rates in men, which is important for identifying HIV-positive men at an early stage in order to link them to appropriate HIV care and treatment services.

### Partner communication crucial for successful HIVST

As summarized in Figure 1, we observed that partner communication was a key factor in shaping the direction that women took in delivering the kits to their male partners. Our findings show that in relationships where there was already good communication about health issues between partners, and where the relationship quality was good, nearly half of the women reported that they delivered the kits on the first or second day after receiving them from the health facilities. However, in relationships where women did not communicate regularly about health issues with their male partners, including in situations where the male partners were not usually around in the home, there were delays in delivering the kits. These observations suggest that partner communication is an important element in enhancing the delivery and eventual use of HIVST kits by male partners [21,22]. However, some of the tactics used by the women, especially the misinformation and failure to provide accurate information, could be potentially detrimental. In relationships where female partners felt apprehensive, some women resorted to using dishonest methods to encourage their male partners to use the kits. Some of these methods included providing partial information about the kit and what its purpose was and lying about the purpose of the kit (e.g. that the kit was for syphilis testing). At least one woman reported that she performed the HIVST process herself. These methods tended to portray elements of coerciveness and could lead to negative consequences or compromise the desired outcomes of HIVST. These findings suggest a need to integrate supportive mechanisms for women in challenging relationships in order to reduce the likelihood of dishonesty and coercive tendencies.

### Female-delivered HIVST acceptable to men

Our findings show that the majority of the male partners were not concerned with receiving HIVST kits from their female partners, although a few were concerned that having the kit delivered by the female partner would put them in a compromised position. If they refused to test for HIV, it is likely that their female partners would think that their refusal is related to their apparent HIV infection or promiscuity status. Nevertheless, our findings reaffirm previous findings from other studies that show that female-delivered HIVST is acceptable and can improve male partner and couples’ HIV testing [2,3,16].

### Adverse consequences following female-delivered HIVST

We did not register any serious adverse events among individuals that self-tested for HIV, including marital violence or suicidal ideation, as feared by participants prior to performing the test. There were more positive outcomes reported, including HIV-status disclosure and partner support to attend antenatal care services, even in relationships where one of the partners was HIV-positive. These findings help to allay anxieties reported in previous studies that HIVST will result in dire social outcomes for the testing partners, and lend credence to the need to promote male partner HIV testing through HIVST.

### Study limitations and strengths

This study had a number of limitations and strengths. The main limitation is that this was a qualitative study in which participants were purposely selected to participate in the study. The purposeful selection of participants does not allow for generalization of our findings. In addition, based on the primary objective of the study, we did not include pregnant women who failed to deliver the kits to their male partners or male partners who failed/refused to use the kits delivered to them by their female partners. Including women who failed to deliver the kits could have yielded insights into the challenges that women experienced in delivering such kits to their male partners. On the other hand, including men who refused to use the kits delivered to them by their female partners could have identified masculinity norms associated with receiving such kits from their female partners which could probably explain why they refused to use the kits. Such data would be important for future interventions aimed at convincing men to test for HIV and to accept HIV self-test kits delivered to them by their female partners. We hope that these insights will be well captured in future studies on this subject.

The strength of this study lies in the fact that besides the study reported by Maman et al. [5], this is the second study to document the processes and strategies that women use to deliver the kits to their male partners and/or convince them to use the kits. We interviewed both HIV-negative and HIV-positive participants, including members of HIV-discordant relationships, and this gave us the opportunity to document perceptions and experiences across the HIV-status spectrum.

## Conclusion

Our findings show that pregnant women used a variety of strategies to deliver HIVST kits to their male partners, some of which bordered on deception. These findings suggest a need to devise strategies to enhance women’s self-efficacy to deliver HIV self-test kits to their male partners as well as improve their communication skills. Study findings show that male partners readily accepted using HIV self-test kits delivered to them by their female partners. This suggests that female-delivered HIVST may help to overcome barriers associated with male partner HIV testing, thereby increasing the proportion of male partners who are aware of their HIV status. Both men and women perceived HIVST as an opportunity to learn about each other’s HIV status, and there were no serious adverse consequences reported from delivering or using the HIVST kits. Our findings lend further credence to previous findings regarding the feasibility of female-delivered HIVST to improve male partner HIV testing in sub-Saharan Africa.

## Paper context

Male partner HIV testing remains low in most settings in sub-Saharan Africa. In this paper, we explored pregnant women and male partners’ perceptions, strategies, and post-test experiences regarding HIVST. We found that women used a variety of approaches to deliver the kits and/or encourage them to use them to test for HIV. Our findings provide additional credence to the feasibility of using female-delivered HIVST kits to improve male partner HIV testing.

## References

[1] Choko AT, Kumwenda MK, Johnson CC, et al Acceptability of woman-delivered HIV self-testing to the male partner, and additional interventions: a qualitative study of antenatal care participants in Malawi. J Int AIDS Soc. 2017;20:1–11. 10.7448/IAS.20.1.21610 PMC5515040 28691442

[2] Thirumurthy H, Masters SH, Mavedzenge SN, et al Promoting male partner HIV testing and safer sexual decision making through secondary distribution of self-tests by HIV-negative female sex workers and women receiving antenatal and post-partum care in Kenya: a cohort study. Lancet HIV. 2016;3:e266–274. 27240789 10.1016/S2352-3018(16)00041-2 PMC5488644

[3] Masters SH, Agot K, Obonyo B, et al Promoting partner testing and couples testing through secondary distribution of HIV self-tests: A randomized clinical trial. PLoS Med. 2016;13:e1002166. 27824882 10.1371/journal.pmed.1002166 PMC5100966

[4] Johnson CC, Kennedy C, Fonner V, et al Examining the effects of HIV self-testing compared to standard HIV testing services: a systematic review and meta-analysis. J Int AIDS Soc. 2017;20:1–10. 10.7448/IAS.20.1.21594 PMC5515051 28530049

[5] Maman S, Murray KR, Napierala Mavedzenge S, et al A qualitative study of secondary distribution of HIV self-test kits by female sex workers in Kenya. PLoS One. 2017;12:e0174629. 28346527 10.1371/journal.pone.0174629 PMC5367822

[6] Choko AT, MacPherson P, Webb EL, et al Uptake, accuracy, safety, and linkage into care over two years of promoting annual self-testing for HIV in Blantyre, Malawi: a community-based prospective study. PLoS Med. 2015;12:e1001873. 26348035 10.1371/journal.pmed.1001873 PMC4562710

[7] Musheke M, Merten S, Bond V. Why do marital partners of people living with HIV not test for HIV? A qualitative study in Lusaka, Zambia. BMC Public Health. 2016;16:882. 27561332 10.1186/s12889-016-3396-z PMC5000425

[8] Matovu JK, Wanyenze RK, Wabwire-Mangen F, et al Men are always scared to test with their partners … it is like taking them to the Police”: motivations for and barriers to couples’ HIV counselling and testing in Rakai, Uganda: a qualitative study. J Int AIDS Soc. 2014;17:19160. 25239379 10.7448/IAS.17.1.19160 PMC4169647

[9] Msuya SE, Mbizvo EM, Hussain A, et al Low male partner participation in antenatal HIV counselling and testing in northern Tanzania: implications for preventive programs. AIDS Care. 2008;20:700–709. 18576172 10.1080/09540120701687059

[10] Byamugisha R, Astrom AN, Ndeezi G, et al Male partner antenatal attendance and HIV testing in eastern Uganda: a randomized facility-based intervention trial. J Int AIDS Soc. 2011;14:43. 21914207 10.1186/1758-2652-14-43 PMC3192699

[11] Mohlala BK, Boily MC, Gregson S The forgotten half of the equation: randomized controlled trial of a male invitation to attend couple voluntary counselling and testing. AIDS. 2011;25:1535–1541. 21610487 10.1097/QAD.0b013e328348fb85 PMC3514892

[12] Carroll JJ, Ngure K, Heffron R, et al Gendered differences in the perceived risks and benefits of oral PrEP among HIV-serodiscordant couples in Kenya. AIDS Care. 2016;28:1000–1006. 26754017 10.1080/09540121.2015.1131972 PMC4917435

[13] Gale NK, Heath G, Cameron E, et al Using the framework method for the analysis of qualitative data in multi-disciplinary health research. BMC Med Res Methodol. 2013;13:117. 24047204 10.1186/1471-2288-13-117 PMC3848812

[14] Braun V, Clarke V Using thematic analysis in psychology. Qual Res Psychol. 2006;3:77–101.

[15] Tong A, Sainsbury P, Craig J Consolidated criteria for reporting qualitative research (COREQ): a 32-item checklist for interviews and focus groups. IntJ Qual Health Care. 2007;19:349–357. 17872937 10.1093/intqhc/mzm042

[16] Matovu JK, Buregyeya E, Arinaitwe J, et al … if you bring the kit home, you [can] get time and test together with your partner’: pregnant women and male partners’ perceptions regarding female partner-delivered HIV self-testing in Uganda - A qualitative study. Int J STD AIDS. 2017;28:1341–1347. 28449628 10.1177/0956462417705800

[17] Spyrelis A, Abdulla S, Frade S, et al Are women more likely to self-test? A short report from an acceptability study of the HIV self-testing kit in South Africa. AIDS Care. 2017;29:339–343. 27654217 10.1080/09540121.2016.1234687

[18] Frye V, Wilton L, Hirshfied S, et al “Just because it’s out there, people aren’t going to use it.” HIV self-testing among young, black MSM, and transgender women. AIDS Patient Care STDS. 2015;29:617–624. 26376029 10.1089/apc.2015.0100 PMC4808283

[19] Makusha T, Knight L, Taegtmeyer M, et al HIV self-testing could “revolutionize testing in South Africa, but it has got to be done properly”: perceptions of key stakeholders. PLoS One. 2015;10:e0122783. 25826655 10.1371/journal.pone.0122783 PMC4380342

[20] Brown AN, Djimeu EW, Cameron DB A review of the evidence of harm from self-tests. AIDS Behav. 2014;18:S445–449. 24989129 10.1007/s10461-014-0831-y PMC4094790

[21] Kumwenda M, Munthali A, Phiri M, et al Factors shaping initial decision-making to self-test amongst cohabiting couples in urban Blantyre, Malawi. AIDS Behav. 2014;18:S396–S404. 24929834 10.1007/s10461-014-0817-9 PMC4102820

[22] Rogers AJ, Achiro L, Bukusi EA, et al Couple interdependence impacts HIV-related health behaviours among pregnant couples in southwestern Kenya: a qualitative analysis. J Int AIDS Soc. 2016;19:21224. 27887669 10.7448/IAS.19.1.21224 PMC5124108

